# Experiences of Mental Health Care Among Women Treated for Postpartum Psychosis in England: A Qualitative Study

**DOI:** 10.1007/s10597-022-01002-z

**Published:** 2022-07-28

**Authors:** Emily Roxburgh, Nicola Morant, Clare Dolman, Sonia Johnson, Billie Lever Taylor

**Affiliations:** 1 Kingston iCope, Camden & Islington NHS Foundation Trust, London, UK; 2 Division of Psychiatry, University College London, London, UK; 3 Health Service and Population Research Department, Institute of Psychiatry, Psychology and Neuroscience, King’s College London, London, UK

**Keywords:** Postpartum psychosis, Postnatal care, Services, Qualitative, Women, Partners

## Abstract

Postpartum psychosis has been found to affect 0.89–2.6 per 1000 women. Onset is typically rapid and severe. Early recognition and appropriate treatment are crucial for a good prognosis. Our aim in this study was to understand women’s experiences of mental health care and services for psychosis in the postnatal period. Semi-structured interviews were conducted with 12 women who reported being treated for postpartum psychosis. Findings were analysed thematically. Women reported that healthcare professionals across maternity and mental health services often lacked awareness and knowledge of postpartum psychosis and did not always keep them or their partners/families informed, supported, and involved. Women wanted better collaboration between and within services, and more efficient, appropriate, and timely care. They valued inpatient services that could meet their needs, favouring Mother and Baby Units over general psychiatric wards. Early Intervention in Psychosis services and specialist perinatal community mental health teams were also well liked.

## Introduction

Psychosis following childbirth usually manifests within the first two weeks after birth (Kendell et al., 1987) and is commonly referred to as postpartum psychosis (PP). PP has been estimated to occur in 0.89 to 2.6 per 1000 women giving birth (VanderKruik et al., 2017). Symptoms include delusions, hallucinations, sleep disturbances, and mood changes (Brockington, 2004). PP can affect women from any background, with many cases occurring unexpectedly in women without a psychiatric history (Sit et al., 2006; Valdimarsdóttir et al., 2009). Risk factors include having a pre-existing mental health diagnosis (especially bipolar disorder [Jones & Craddock 2005]), having had a prior episode of PP, obstetric complications, immunological factors, sleep deprivation, and psychosocial factors (Jones et al., 2014; Isik, 2018). There is a lack of consensus regarding the classification of PP. Currently, the ICD-11 (World Health Organization, 2019) and the DSM-5 (American Psychiatric Association, 2013), do not classify PP as a distinct diagnostic entity. Nonetheless, the term PP remains common in clinical use (Jones & Craddock, 2005; Di Florio et al., 2013). PP is typically seen as a time of “psychiatric emergency” which often results in hospitalization (Heron et al., 2008). Continued poor maternal mental health can disrupt the parent-infant bond and have long-term implications for women, their babies, and partners/families (Blackmore et al., 2013; Forde et al., 2020; Jones et al., 2014). Early and effective treatment are considered crucial to achieve the best possible prognosis (Boyce & Barriball, 2010). With appropriate treatment, PP has generally good long-term and functional recovery outcomes (Burgerhout et al., 2017; Rommel et al., 2021). However, women remain at an increased risk for future psychiatric episodes (Robertson et al., 2005).

In the UK, and some other countries such as Australia and France, specialist Mother and Baby Units (MBUs), which allow joint admission of the mother and her infant, are viewed as best practice (Connellan et al., 2017). However, geographical disparities and small numbers of beds are common barriers to accessing specialist perinatal support. In many countries, MBUs are not available at all. In the UK, a recent expansion of specialist perinatal mental health services means there are now 22 MBUs across the country, along with specialist community perinatal teams in all localities. However, some women are still admitted to general psychiatric wards, which involves separating them from their babies, or women may receive treatment in the community from non-perinatal mental health teams such as Crisis Resolution Teams (CRTs; multidisciplinary general mental health teams designed to support adults experiencing a mental health crisis to remain at home) (Johnson, 2013), or Early Intervention in Psychosis (EIP) services (multidisciplinary community services offering ongoing support to people experiencing a first episode of psychosis) (Birchwood et al., 2000). Treatment in the community is often in addition to prior and or subsequent inpatient admission. Some women and partners prefer to avoid hospital stays and be seen by CRTs at home (Khalifeh et al., 2009), however, CRTs often lack specialist perinatal mental health expertise and may struggle to manage perinatal psychosis unless they collaborate effectively with specialist services (Rubio et al., 2021).

There has been limited research to date exploring experiences of care among women treated for PP. One study found that women were often dissatisfied with staff who cared for them, especially in relation to their general postnatal care (Engqvist et al., 2011). Other women report feeling frustrated with healthcare services because of a lack of information and support, especially for those treated in general adult psychiatric services rather than MBUs (Robertson & Lyons, 2003). Mothers find admission to general psychiatric units distressing and inappropriate for their needs, but often struggle to get access to specialised perinatal support (Doucet et al., 2012; Heron et al., 2012; Forde et al., 2019; Griffiths et al., 2019). Knowledge of PP among healthcare professionals and information provision have also been found to be poor (Doucet et al., 2012; Heron et al., 2012; Dolman et al., 2013). So far, few studies have used a qualitative design to explore in-depth women’s experiences of current service systems and the gaps and unmet needs identified. In the current study we aim to address this gap in the literature by exploring how women with PP experience services and formal postnatal support, and what kind of care they would prefer. Exploration of experienced and perceived shortfalls of this kind can contribute to underpinnings for service improvement and development of models of specialist care for PP, helping to improve outcomes.

## Methods

This study was part of a wider qualitative study (known as the STACEY study), exploring experiences of a range of services treating women diagnosed with a variety of perinatal mental health conditions. The STACEY study was part of a larger research programme called Effectiveness of Services for Mothers with Mental Illness (ESMI). NHS ethics approval was obtained (reference: 13/LO/1855).

## Participants

Twelve women from the wider project (of 52 women) who all self-reported having experienced PP were included in this study. Eight out of 12 women had a pre-existing severe mental health diagnosis of either schizophrenia, bipolar disorder, or affective psychosis; their characteristics are further described in the Results section. Women were recruited from six healthcare providers covering a range of areas across England. For the wider STACEY study, women were purposively sampled to obtain diversity of diagnosis, service use, and socio-demographic background. Inclusion criteria required that women were 16 years or over; English language speakers; had accessed NHS treatment for a perinatal mental health difficulty during or after their most recent pregnancy; and had a baby aged 6–9 months old. Eligible women were identified and approached by a clinician within their mental health team. Those expressing an interest in participating were contacted by a researcher to provide them with more information about the study. Individuals lacking capacity to consent were excluded. Participants were informed that their contributions would be kept confidential, with anonymity of quotations ensured. Interviews were carried out between 2015 and 2017. Informed written consent was obtained.

## Data Collection

Interviews were semi-structured, using an interview schedule containing open questions to guide areas of discussion. This explored women’s views of services they accessed for their perinatal mental health across their entire care pathway, as well as their partner’s involvement in their care. Questioning was flexible and responsive to women’s replies. Interviews lasted approximately one hour, usually in participants’ homes. The interview schedule was developed by the research team (academics and clinicians with specialist expertise in perinatal mental health) and was reviewed by a perinatal service user and carer panel. It was piloted with one woman from the perinatal service user and carer panel (diagnosis PP), and four women who had accessed perinatal mental health support. Most interviews (n = 11) were conducted by the main STACEY researcher (a clinical psychologist and mother). One interview was carried out by an MSc student with the main researcher present.

## Analysis

Interviews were audio-recorded, transcribed, anonymised and imported into NVivo 12 Software. Thematic analysis (Braun & Clarke, 2006) was carried out by the first author. An inductive approach was used to explore commonalities and variations in the experiences of women’s postnatal care. Each transcript was read and reread to identify initial codes which were then developed into themes using data from across the sample. Themes were refined and restructured as analysis progressed. To enhance rigour, a second researcher analysed a subsample (n = 3) of the interviews independently, and the main STACEY researcher (BLT) read all transcripts. Regular discussions among members of the research team were held throughout the analytic process, focusing on interpretations of the data and revisions of the initial thematic framework. As the importance of reflexivity is emphasized in qualitative research (Berger, 2015), researchers made regular efforts to question the influence of their backgrounds and beliefs on the interpretation of data and generation of themes.

## Results

### Participant Characteristics

Women’s characteristics are shown in Table 1. Their mean age was 35 (range: 27 to 43). Most women were highly educated, from a white British background, and living with a partner. Eight out of 12 women had a pre-existing severe mental health diagnosis and prior contact with mental health services. Of the remaining four women, two had no prior contact with mental health services and two had accessed support for depression. There was variability in the mental health services accessed by women, and thus their experiences of care were diverse (see Table 1). Using multiple forms of postnatal mental health care is frequent. Table 2 displays the details of the key services women had accessed; they could be supported by more than one service at different time points. Of the 12 women, eight were hospitalized and one was admitted to a crisis house; of the remaining three, one turned down an MBU admission and was seen by a perinatal community mental health team, while two accessed EIP services in the community and were not hospitalized. Overall, seven women accessed either a specialist MBU or specialist perinatal community mental health team.

**Table 1:** Characteristics of women (N = 12)

Characteristics		Category	Respondents Mean or N
Mother’s age (years)		Mean Age	35
Ethnicity		White British	7
		White other	1
		Asian	2
		Mixed – Black African/White	1
		Black Caribbean	1
Level of education		Postgraduate degree or above	7
		Higher Education Diploma/ Bachelor’s degree	2
		A-Levels	1
		GCSE	2
Living with partner		Yes	11
		No	1
Number of children		1	6
		2	4
		3+	2
Previous service use for mental health		Yes	10
		No	2
Services used in the postnatal period	Perinatal	Mother and baby unit	6
(Women could access more than one service)		Perinatal community mental health team	4
	Non-perinatal	General acute psychiatric ward	5
		Crisis house	1
		General community mental health team	5
		Early intervention in psychosis services	3
		Crisis resolution team	3
Psychiatric status (self-reported)		Pre-existing bipolar disorder	6
		Pre-existing schizophrenia	1
		First onset of postpartum psychosis	4
		Second onset of postpartum psychosis	1

### Overview of Qualitative Findings

Six key themes were identified in the analysis: (1) the influence of maternity care on women’s mental health, (2) lack of knowledge, awareness and information on PP, (3) problems accessing care, (4) inpatient admissions: the need for a therapeutic environment, (5) value of consistency, cohesiveness and choice and (6) being mindful of the partner.

**Table 2:** Details of mental health services accessed by women

Specialist perinatal or non-perinatal service	Type of service	Description
Specialist perinatal	Mother and baby unit	Hospital that admits the mother alongside her baby and provides tailored support for serious mental health problems in postnatal period
	Specialist perinatal community mental health team	Multidisciplinary teams treating women in the community with complex postnatal mental health difficulties
Non-perinatal	General acute psychiatric ward	General psychiatric hospitals for adults with mental health difficulties where women are admitted without their baby
	Crisis house	General short-term community based housing for adults with mental health difficulties where women are admitted without their baby
	General community mental health team	Multidisciplinary teams supporting adults with mental health difficulties
	Early intervention in psychosis services	Multidisciplinary teams offering on-going support to people experiencing a first episode of psychosis
	Home treatment team/crisis resolution team	Multidisciplinary teams offering short-term home treatment to people experiencing an acute mental health crisis

### The Influence of Maternity Care on Women’s Mental Health

It was conspicuous that many women reported experiencing physical difficulties related to pregnancy and childbirth (e.g., postpartum haemorrhages, preeclampsia, retained placenta), as well as problems with their maternity care. Some explicitly connected poor maternity care and a stressful hospital environment to the emergence of their mental health difficulties. Examples of poor maternity care were women not being allowed to stay in hospital when they felt they needed support, delays being attended to during labour, and difficulties getting adequate care from midwives and other professionals in hospital. Sleep deprivation was common. One mother related this to the disruptive, noisy, and overheated hospital environment and felt that sleep deprivation had precipitated the onset of hallucinations.

It was atrocious. The level of care at [the hospital] was ****** appalling to be honest. And I think it was probably that that triggered it and I mean [my perinatal mental health nurse] said it was probably that that triggered it because I had been absolutely fine (P01).

Women reported that maternity hospital staff did not seem to be aware that mental health could deteriorate rapidly following a traumatic or difficult birth. This was particularly important for women who had a pre-existing diagnosis, where they felt that midwives were not always aware of their mental health history and therefore did not class them as high risk for postpartum difficulties. Even when classed as high-risk, some women felt that staff did not follow up to ensure their mental health was assessed before discharge from hospital after giving birth.

Nothing was followed up…When I had the baby, I heard [the specialist mental health obstetrician], she was in the hospital, and she didn’t come and see me. And I just thought that was a bit poor really…And my usual consultant said, ‘Oh, we’ll refer you to the liaison psychiatry team at the hospital’… But she kind of said it every time, put it in my notes…I never heard from them (P07).

It was common for women to report that they found some maternity professionals helpful and supportive, but others were felt to lack empathy and proactiveness. One woman felt that hospital staff “didn’t really get that I was mentally and physically exhausted” (P05), while another reported that midwives: “were just really stretched… I hardly got to have a word with a proper midwife” (P11).

### Lack of Knowledge, Awareness and Information on PP

Women with pre-existing mental health diagnoses described variable experiences of healthcare professionals providing information about the potential risks of childbirth on their mental health. Only a few women said that they were informed about PP and what to look out for. Others were either not informed, were told that pregnancy could serve as a protective factor, or were advised that they were unlikely to experience PP.

She [my perinatal psychiatrist] said that there’s a one in five chance of you getting [PP] again, which she should’ve said one in two. And she also said to me, which I thought was really unhelpful although she was trying to be helpful, was that she’s seen lots of people and no one has ever had it again, no one…So when I got it I felt I failed. (P04)

These experiences resulted in some women feeling unprepared and sometimes delaying help-seeking that, in their view, worsened the experience and severity of the PP episode.

I would have…much preferred to be prepared…read up on it, had a leaflet on-, this could happen to you, just make sure you get enough sleep, make sure if you need to increase your medication-… I would have been a bit more aware and spoken to the psychiatrist sooner and got help sooner… I just wasn’t very prepared (P03).

The four women in this study who did not have an existing mental health diagnosis had not received any information at all regarding PP. For them, PP was unheard of; the only disorder related to pregnancy and childbirth that they knew of was postnatal depression. Receiving a PP diagnosis could be frightening for some women.

I didn’t even know what psychosis was until I got diagnosed. And I was like, really? I’ve never heard of it. Psychosis, psychotic just makes me think of a mad murdering man that’s going around killing everybody. Like that is what I thought of when they told me I had that. I was absolutely shocked… but I’m just a normal woman, just had a baby (P05).

Women emphasized the need for more training and information around the presentation of PP, especially among midwives, due to their ongoing contact with women throughout their pregnancies. They felt that some healthcare professionals only appeared to be aware of postnatal depression.

They kept treating me like I was just depressed… but it was something more than that…but nobody seemed to listen to me. So I do think that they could all do with...a bit of training or even just an information booklet to read (P05).

### Problems Accessing Care

Women had diverse experiences of accessing mental health care postpartum, with the involvement of many different healthcare professionals playing a part in this. Several women reported having to call or attend the Accident and Emergency department (A&E), either on their own initiative or because they had spoken to someone supporting them (e.g., a health visitor) and were advised to do so. Experiences of waiting in A&E were not always positive and women could be left “sat in the waiting room for I don’t know, hours…getting worse and worse…I was hallucinating” (P11).

A few women accessed mental health services via their GP and described mixed experiences of this. One woman subsequently waited two weeks until reaching crisis point, which was followed by an MBU admission, due to her husband’s initiative in calling an ambulance. A couple of women reported that they felt fortunate to be referred to an EIP service, as they found these teams proactive and accessible.

Several women who were hospitalized stayed on an acute psychiatric ward prior to an MBU admission, as there were no available beds. Other women described a quicker process of being admitted into an MBU. Women who were particularly proactive themselves, had assertive and or well-resourced partners or family networks helping them access support, or who had a proactive healthcare professional already involved in their care (those with pre-existing diagnoses), seemed to access support quicker.

We decided to try and find a Mother and Baby Unit because things were sort of progressively worsening quite quickly… we contacted her [my care coordinator] as soon as we could …she said that she would try and get a bed and initially they came back and said that there wasn’t anything and then I started to get really desperate…So she tried again and then managed to find me a bed… (P08).

### Inpatient Admissions: The Need for a Therapeutic Environment

Most women in our sample had been hospitalized on MBUs and or acute wards. It was common for women to describe MBUs as being clean, with attractive grounds, and good rooms.

We took some pictures because it’s in a garden and whenever we show someone, they’re like ‘where’s that beautiful park?’. So it’s as if it’s like not some mental health-…It was so beautiful…That’s why I say it felt like a spa because it felt so-…it felt quite luxurious (P04).

In contrast, acute psychiatric wards were not considered to be an appropriate environment for a woman who had just given birth, especially in the context of PP. For example, one woman had needed breast-pads, but these were not readily available (P08). Others felt confused about being separated from their baby, which they felt exacerbated their PP symptoms. There was an overall dislike of acute ward facilities, with some women linking them to prisons. A lack of cleanliness, privacy, and clear layout of the wards caused women to feel distressed and or frightened.

It just felt horrible…I was just really confused about the whole layout of the place… The toilet was horrible, the shower was horrible, the condition of the place was not very nice… post birth you need…clean sanitation...so I was bleeding a lot still…I needed a bath but you don’t have baths in your room (P11, acute ward and MBU).

Similar contrasts emerged in descriptions of the support provided in these in-patient settings. Women found MBUs comfortable and described how they found the practical support very helpful (e.g., learning how to feed, change, and wash their baby). However, those who were in general acute wards did not have the benefit of such support. Another aspect of services was the available activities (e.g., swimming, arts and crafts, etc.) and interactions. On MBUs, women welcomed the chance to choose to do activities with or without their baby. MBUs also allowed women to interact with other mothers going through similar situations, so they were able to share experiences and insight. However, this was not available to women in acute wards.

I don’t know if I was the only mum in [the acute ward]. I didn’t really talk to the other patients that often. But the MBU [which I went to afterwards] had things like OT [occupational therapy] …As far as I’m aware they had nothing at the acute psychiatric ward, it was just inside. I don’t remember going outside. I just remember being in my room or being in the canteen (P03).

### Value of Consistency, Cohesiveness and Choice

Over half of the women in the study emphasized the importance of having a clinician involved throughout their care who was consistent and readily available when support was needed. Consistency was particularly important given the nature of PP, as some women described how having different people involved increased their paranoia and made it difficult to trust professionals. CRTs were reported not to have consistent staff making home visits, however, EIP services and perinatal community mental health teams were well-liked.

I had a [Community psychiatric nurse (CPN)] come around every week [from the EIP service] or more often if I needed it, or telephone conversations…she was regularly available…things were dealt with quite there and then and efficiently (P12).

Although a few women stated that it could be helpful interacting with different healthcare professionals (even from different services) to gain alternative perspectives, sometimes this would lead to many different opinions and information being provided, leaving some women feeling anxious and unsure, especially with regards to advice caring for their baby.

There were so many people involved, and they just all said different things and there were people worried about different things and I just felt kind of really worried about how it was going to be. And I think they made it worse. I think if I’d just seen the same person…A bit of consistency (P07).

Women were sometimes reliant on others (partners and healthcare professionals) to help them make decisions because of their difficulties, which made consistency particularly important for building rapport. Nearly all women valued inclusion in decisions regarding their care – for example, around medication, who was involved in their care, and the type of service they wanted to access. This was particularly important given the lack of control women expressed whilst experiencing PP. Their accounts suggested that MBUs and EIP services made more efforts to involve women in decisions compared to acute wards and CRTs.

### Being Mindful of the Partner

Most women recognised the challenges the postnatal period could have for partners, fathers, and other family members, especially in the context of PP. Some women valued their partners being provided with information, support and being involved in decisions about their care.

He is very much involved, they [the MBU] try to keep him up to date because it’s obvious that they see that I couldn’t really make the decisions on my own… they give him information as well as myself…they treat us as equal partners (P10).

Women who were not hospitalized reported that healthcare professionals often did not involve their partners unless women themselves made the effort to include them. In comparison to community care and acute wards, women described how MBUs offered partners more opportunities to attend weekly reviews or scheduled appointments, although sometimes women felt there was still room for improvement. Healthcare professionals would take this opportunity to keep family members informed and seek their opinions on how they thought the mother was doing. Some partners were not able to attend due to work commitments, childcare, or the long home-MBU distance. Acute wards made fewer efforts to involve partners.

Yes, I think the MBU were better at communicating and getting him involved and doing things…I mean I don’t remember too much about the acute psychiatric ward, but it was nearer for him. I just think the set up wasn’t as good…I think much more organised at the MBU (P03).

Most women reported that their partners were not offered any support themselves, and a few women stated that their partners would not want it. However, women also felt that help was only tailored for them, as mothers, and not for their partner. Only one woman reported that her partner was offered to speak to someone informally at the MBU, but he felt unable to take this offer up in practice as the staff seemed too busy (P04).

## Discussion

We aimed in this study to gain insight into experiences of care in the postnatal period amongst women with PP. Most women described a distressing and unaccommodating maternity hospital experience during and following childbirth and, on occasion, connected these challenges to the manifestation of their PP episodes. In line with previous research, this appeared more likely when in combination with difficult births (Jones et al., 2014). It has been reported that women value being informed about PP during their perinatal care (Robertson & Lyons, 2003; Engqvist et al., 2011; Heron et al., 2012). However, many women in this study were not informed of PP, even when they had a pre-existing mental health diagnosis. Similarly, as highlighted in previous research (Engqvist et al., 2011), women in this study thought that healthcare professionals should receive more training about PP, especially midwives, who have ongoing contact with women during pregnancy, birth and beyond.

It has been well documented that women presenting with PP require urgent attention (Doucet et al., 2012; National Collaborating Centre for Mental Health, 2018), however not all women in our study felt they got support rapidly. Delays due to lack of awareness or initiative among healthcare practitioners like GPs and midwives was a common barrier, confirming previous reports that GPs feel unprepared for dealing with perinatal mental health conditions (Khan, 2015). Women typically described difficulties accessing MBUs and or sometimes being admitted to acute wards first or instead, although this may have improved since the recent expansion of specialist MBUs in the UK (NHS England, 2016). Building upon previous research, there was a clear preference for MBUs in comparison to general acute psychiatric wards (Heron et al., 2012; Wright et al., 2018; Griffiths et al., 2019). Several women reported that acute wards were less comfortable and described them as confusing in the context of PP, and inappropriate for a woman experiencing postnatal physical challenges. MBUs were viewed as safe, tailored to their needs, and as offering more support relating to both being a new mother and to mental health issues. These findings are in keeping with Howard et al.’s (2022) finding from the quantitative arm of the ESMI study that satisfaction is greater for MBUs than for general catchment area inpatient or CRT care, although the same study did not find a difference in readmissions for types of acute care.

Continuity of care and strong therapeutic relationships with staff are vital for building trust, maintaining stability, and improving recovery (Sweeney et al., 2014). Inadequate staffing levels, disorganisation, and lack of staff proactiveness and empathy have been reported to disrupt this (Bassett et al., 1999; Wood et al., 2016; Nagle & Farrelly 2018), consistent with the findings in this study. Some women felt that having too many people involved in their care led to increased confusion, paranoia, and distrust, characteristics that are already common in PP (Heron et al., 2008). Women with PP often report feelings of ‘lacking control’ in many aspects of their lives (Robertson et al., 2005). This was an issue for women in this study, who felt they sometimes had to rely on healthcare professionals and or their partners to make decisions for them, but also saw it as vital that they were included in treatment decisions to feel a sense of regaining control. Women felt EIP services were well-tailored to meet their needs, even though they are not a specialist perinatal service. Likewise, MBUs and perinatal community mental health teams were also favoured. Reports were less positive for other generic non-perinatal mental health services, such as CRTs (Rubio et al., 2021).

Partner involvement was valued by women, especially when their PP episodes deteriorated and they felt reliant on their partners, as may have especially been the case where women have lost capacity to make decisions during an acute psychotic episode, or where partners were required to provide most childcare. In line with previous research (Lever Taylor et al., 2018, 2019a), MBUs were seen as the most inclusive (in comparison to other services) for both involvement and support offered, although there was still room for improvement. This reinforces previous research which has shown that partners are often marginalised across services, and that there is a need to consider how to involve family members where possible, while also retaining a clear focus on the needs of women and their autonomy (Lever Taylor et al., 2019a).

## Strengths and Limitations

While this study provided unique insights into care experiences in the postnatal period amongst women with PP, it also had several limitations. Firstly, most of the women included were white British, with higher-than-average educational levels, and our findings may not be transferable to mothers from other backgrounds. Although these characteristics are overrepresented in this study and a sample size of 12 is relatively small, the prevalence of PP makes larger-scale studies challenging. Similarly, eight out of 12 women had a pre-existing severe mental health diagnosis and prior contact with mental health services, thus findings might say less about the experiences of women who experienced PP without any previous mental health diagnosis and or contact with mental health services. Perinatal mental health services have been expanding in the UK; therefore, views of staffing and service provision may have changed in some areas since we carried out these interviews. Likewise, we describe experiences within the service configurations characteristic of England and at a time when under-funding and scarcity of resources where widely reported; generalizability beyond the UK may be limited.

## Clinical Implications

This study’s findings suggest varied and complex care experiences for women experiencing PP, revealing some of the difficulties that poor provision of specialist perinatal mental health services and lack of perinatal mental health knowledge among healthcare professionals can cause. Findings suggest that, together with recent investments in specialist perinatal services in the UK (NHS England, 2016), additional training on perinatal mental health problems for general psychiatric ward and or non-specialist community mental health teams, GP’s and maternity staff in hospitals is warranted. This should include basic information on the differences between postnatal depression and PP and understanding women’s particular needs in the context of PP, especially relating to continuity of care and risk assessments, and the importance of including partners/ family in treatment (Lever Taylor et al., 2019b). ‘Perinatal champions’ within services could facilitate and promote this. Positive experiences of EIP services, together with the reported importance for women of staff continuity and consistency, suggest that these services may offer better continuity over a longer period than a specialist mental health team. Joint working with perinatal teams may be a means to ensure women with psychosis following childbirth benefit from both forms of specialist care. Similarly, closer liaison between and within services is fundamental to reducing barriers and confusion for these women. There is also a need to consider necessary adaptations to general psychiatric ward environments to better cater for women following childbirth.

Lastly, it is important to consider inequalities of accessing postnatal care. Most women in this study were highly educated and white British, and there were suggestions from findings that women with greater ‘social capital’, including proactive, confident and well-resourced family and social set-ups, may have been better able to facilitate quick access to services. This is especially important as there is evidence that women from more disadvantaged or socially excluded groups may have greater difficulty accessing support in the perinatal period and may have poorer experiences of care (Moore et al., 2019; Smith et al., 2019; Watson et al., 2019). Future research could explore the needs of these women in greater depth to better inform these inequalities of access in the context of PP.

## Conclusions

This study highlights the importance of considering what is happening externally (in the wider context of care), as well as internally (e.g., biological, hormonal, psychological, or pre-existing risk factors) for women with PP. Attention should be paid to maternity care and the hospital environment, as these are modifiable factors that could ensure women’s needs are better met at an early stage. It is necessary to provide accurate information and training to healthcare professionals regarding PP and encourage them to share this information with women and their partners throughout the perinatal period. This could improve timely identification and effective treatment delivery. Finally, there was an overall preference for MBUs and community perinatal mental health teams, supporting the current UK increases in investment in specialist perinatal mental health services (NHS England, 2016).

## Data Availability

Data and materials are not available for distribution to others than the research team.

## Declarations

### SRQR guideline

Followed.
